# Simultaneous QTL detection and genomic breeding value estimation using high density SNP chips

**DOI:** 10.1186/1753-6561-4-s1-s9

**Published:** 2010-03-31

**Authors:** Roel F Veerkamp, Klara L Verbyla, Han A Mulder, Mario P L Calus

**Affiliations:** 1 Animal Breeding and Genomics Centre, ASG Wageningen UR, PO Box 65, 8200 AB Lelystad, The Netherlands; 2 Biosciences Research Division, Department of Primary Industries Victoria, 1 Park Drive, Bundoora 3083, Australia

## Abstract

**Background:**

The simulated dataset of the 13^th^ QTL-MAS workshop was analysed to i) detect QTL and ii) predict breeding values for animals without phenotypic information. Several parameterisations considering all SNP simultaneously were applied using Gibbs sampling.

**Results:**

Fourteen QTL were detected at the different time points. Correlations between estimated breeding values were high between models, except when the model was used that assumed that all SNP effects came from one distribution. The model that used the selected 14 SNP found associated with QTL, gave close to unity correlations with the full parameterisations.

**Conclusions:**

Nine out of 18 QTL were detected, however the six QTL for inflection point were missed. Models for genomic selection were indicated to be fairly robust, e.g. with respect to accuracy of estimated breeding values. Still, it is worthwhile to investigate the number QTL underlying the quantitative traits, before choosing the model used for genomic selection.

## Background

High density SNP chips with ~50,000 SNPs have become available for most livestock species. Breeding value estimation using all these SNPs simultaneously is expected to yield the highest accuracy [1]. Several parameterisations of the SNP effects in the statistical model have been suggested [2-5]. The objectives of this study were to accurately identify QTL and predict breeding values in the simulated data of the 13^th^ QTL-MAS workshop, using different parameterisations for the SNP effects.

## Methods

The simulated data of the 13^th^ QTL-MAS workshop is described Coster et al. [6]. Simulated data were analysed per time point, and for QTL detection, the change between traits at subsequent time points was also used. A pedigree based model was fitted using ASREML [7]. The Gibbs sampler described initially by Meuwissen and Goddard [1] and Calus et al. [4,5] was used for models including the SNP parameterisations. The general model used was:

, where y_i_ is the phenotypic record of animal i, µ is the average phenotypic performance, animal_i_ is the random polygenic effect for animal i, haplotype_ijk_ is a random effect for a paternal (k = 1) or maternal (k = 2) haplotype at locus j (of nloc loci) of animal i, and e_i_ is a random residual for animal i. The first parameterisation was a simple BLUP model with the additive relationship matrix between the animals only. Other parameterisations assumed the SNP effects came from one distribution (SNP1), i.e. BayesA, from two distributions (SNP2 i.e. BayesC), or from three distributions allowing for small, medium and large SNP effects (SNP3). A further parameterisation assumed a QTL was placed in between two SNP and 453 IBD matrices were calculated for all the haplotypes at a bracket using linkage disequilibrium and linkage analysis information [2]. Finally, a parameterisation used the phased genotypes to construct identical by state haplotypes from either 2 or 5 SNP, (IBS2 and IBS5, respectively) as presented before by Villumsen et al. [3] but with the addition that the same SNP were used at the border of two neighbouring brackets. The final reduced model included the 14 selected SNP that had a posterior probability >0.1 of affecting a QTL in the SNP2 analysis.

## Results

### Pre-analysis

An important question is how to model the time series data, and extrapolate the breeding values to the required time point 600. The mean of the traits indicated that points 265, 397 and 530 are in the linear part of the growth curve, confirmed by high phenotypic, and genetic correlations between those points (> 0.95). Graphical inspection confirmed that little information was available to estimate the inflection point or asymptotic values at population individual or genetic level. Therefore all five time point were analysed separately and linear regression fitted through the breeding value at point 265, 397 and 530 was used to extrapolate breeding values to the required point 600.

### QTL detection

**Figure 1:**
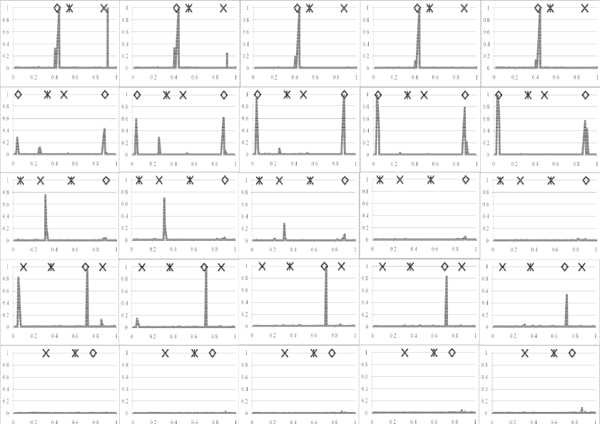
**Posterior QTL probabilities using SNP2 model. Columns from left to right are time points 0, 132, 265, 397 and 530 respectively, and rows from top to bottom are chromosomes one to five.** Y-axis is posterior probability (scale 0 to 1) and X-axis is location on each chromosome in M (scale 0 to1). Simulated QTL are indicated by ◊, ж and x for asymptote, inflection point, and relative growth rate respectively.

**Figure 2:**
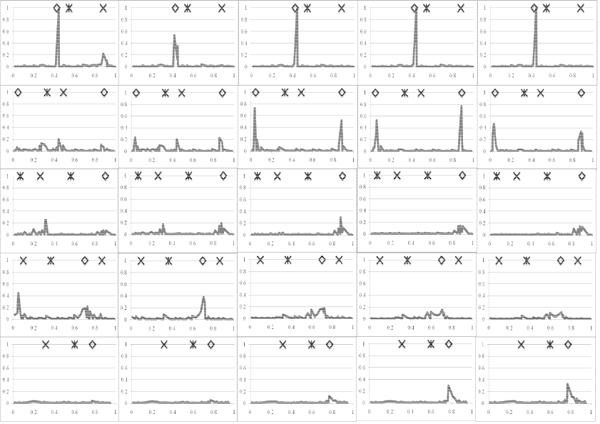
**Posterior QTL probabilities using IBD model. Columns from left to right are time points 0, 132, 265, 397 and 530 respectively, and rows from top to bottom are chromosomes one to five.** Y-axis is posterior probability (scale 0 to 1) and X-axis is location on each chromosome in M (scale 0 to1). Simulated QTL are indicated by ◊, ж and x for asymptote, inflection point, and relative growth rate respectively.

In total 14 SNP had a posterior QTL probability above 0.10 for at least one of the time points (Figure 1). For example, on chromosome one at position 0.4447 a strong QTL was found affecting the trait at each time point and the change in traits between time points, independent of the model used for analysis. The SNP2 model gave QTL also at locations 0.4029 and 0.9137 on chromosome one. The latter clearly affected the trait at time point 0, had little effect at point 132, and had no effect thereafter or on the change of the trait between the time points. The IBD model distributed this QTL effect across a few more SNP (Figure 2), leading to a lower maximum posterior probability around location 0.9137. This lower posterior probability spread across more brackets was generally observed for the IBD model compared to the SNP2 model.

### Breeding values

Table 1 gives the correlations between the breeding values (for animals without phenotypic information) predicted with the different parameterisations. Correlations were high between most models that included the SNP information. Albeit the breeding values from the model assuming that all SNP effects came from one distribution (SNP1) differed. Even the analysis including only the 14 SNP selected on the basis of the posterior probability >.10, gave correlations close to unity with the more extensive models. Similarly correlations with true breeding values were 0.93 and 0.92 for all SNP models and 0.91 for the SNP1 model (Table 1), respectively. Overall predicted breeding values appeared insensitive to the models used.

**Table 1:** Evaluation of predicted breeding values (EBV) at point 600 for the animals without phenotypic data

	BLUP	SNP1	SNP2	SNP3	IBS2	IBS5 IBD	14 SNP
**BLUP**	1						
**SNP1**	0.71	1					
**SNP2**	0.76	0.93	1				
**SNP3**	0.75	0.93	1	1			
**IBS2**	0.74	0.93	0.99	0.99	1		
**IBS5**	0.75	0.94	0.99	0.99	0.99	1	
**IBD**	0.75	0.95	0.98	0.98	0.98	0.98 1	
**14 SNP**	0.72	0.93	0.99	0.99	0.99	0.98 0.97	1

	**Association EBV with true breeding value**					

**Variance EBV**	10.8	25.0	18.0	17.9	18.3	19.6 19.0	20.2
**Accuracy**	0.65	0.91	0.93	0.93	0.93	0.93 0.93	0.93
**Mean sq. error**	14.7	4.4	3.8	3.7	3.5	3.5 3.5	3.5
**Regression**							
**coefficient**	0.99	0.92	1.10	1.10	1.10	1.06 1.08	1.04

Correlations between breeding values predicted using the additive genetic relationship matrix (BLUP), and haplotype defined as single SNP (effects sampled from 1, 2 or 3 distributions), IBD haplotypes, and IBS haplotypes (combing 2 or 5 SNP), and association with simulated true breeding value.

## Discussion

Using all SNP simultaneously, 14 QTL were identified with relative sharp peaks in posterior probability and 9 of these were within 5 cM of the 18 QTLs simulated, and all 14 were within 10 cM. Surprisingly few false positive QTL were found especially since the cut off point for the posterior probability of 10% was set arbitrarily. In the context of the simulated growth curve model, five QTLs were found for the asymptote, and four were close to the simulated QTL for relative growth. In our analysis these QTL for relative growth rate were found at the first time points only, as expected since here the effect is largest on the variance. As suggested by the preanalysis no QTL was found within 5 cM of the QTL affecting the inflection point, albeit on chromosome 2 one QTL was close. It would be interesting to see if using the growth model in the analysis would be more successful in picking up the QTL for the inflection point, since such a model resembles the underlying simulated model closer and requires two parameters less to be estimated, compared with the model used here. The disadvantage of fitting the growth curve model might be that sampling covariance between the three parameters, together with the inability to separate these parameters in the current data, might lead to more spurious QTL estimates. 

Little difference was found between the IBD and SNP methods, although some of the peaks were distributed across more SNP when using IBD. This might be linked to the genetic history of the QTL or with the parameterisation. For example when the QTL is fixed at a SNP, then using brackets of two SNP will split the effect across the two brackets.

From the correlations and the MSE the breeding values appear fairly robust across the different models with the exception of the model assuming that all SNP effects can be captured with one distribution. The exception of model SNP1 is because the assumption on the distribution of the SNP effects is violated, because some large QTL were present and most SNP had no effect in the simulated data. Interesting to observe that, apart from the BLUP analysis, all regression coefficients deviated from one (Table 1). SNP1 smaller and the other models above one, we have no explanation for this difference. The analysis including a subset of 14 SNP gave high correlations with the other fully parameterised methods, suggesting there was considerable scope in reducing the number of SNP required when the QTLs were estimated in this dataset. However, this is in agreement with findings in real data also [8].

## Conclusions

Nine out of 18 QTL were detected, however the six QTL for inflection point were missed. Models for genomic selection were indicated to be fairly robust. Still, it is worthwhile to investigate the number QTL underlying the quantitative traits, before choosing the model used for genomic selection

## Competing interests

The authors declare that they have no competing interests.

## Authors' contributions

RFV carried out the analyses and drafted the manuscript. MPLC developed the software and together with HAM and KV helped to interpret the results and present them in the manuscript. All authors read and approved the final manuscript.
